# Sports and physical activity interventions in autism spectrum disorder: a systematic literature review and meta-analysis

**DOI:** 10.3389/fspor.2026.1793290

**Published:** 2026-06-26

**Authors:** Udeme Samuel Jacob, Sri Nurhayati, Mbulaheni Obert Maguvhe

**Affiliations:** 1 Department of Inclusive Education, School of Educational Studies, College of Education, University of South Africa, Pretoria, South Africa; 2 Department of Community Education, Postgraduate Program, Institut Keguruan dan Ilmu Pendidikan Siliwangi, Cimahi, Indonesia

**Keywords:** autism spectrum disorder, children and adolescents, executive function, intervention, meta-analysis, motor skills, physical activity, social communication

## Abstract

**Background:**

Physical activity (PA) interventions have been explored as a means of improving motor, social, executive, behavioural, and psychosocial outcomes in children and adolescents with autism spectrum disorder (ASD). However, variability in study designs and methodological quality raises questions about the consistency and reliability of reported effects.

**Methods:**

A systematic review and meta-analysis were conducted, including studies published between 2019 and 2025. Eighteen studies met the inclusion criteria for qualitative synthesis, and twelve were included in the meta-analysis using a random-effects model. Effect sizes were calculated and pooled across outcome domains. Risk of bias was assessed, and additional analyses were conducted to examine heterogeneity and potential publication bias.

**Results:**

PA interventions were associated with improvements across multiple domains. The pooled effect size was large (*d* ≈ 1.28), but substantial heterogeneity across study designs, intervention types, and sample characteristics was observed. Subgroup analyses indicated variation in effects across intervention types and participant groups, though findings were not consistently stable. Risk-of-bias assessments identified several methodological limitations, and publication bias analyses suggested possible asymmetry. These factors indicate that the magnitude of the pooled effect size may be overestimated.

**Conclusion:**

PA interventions show promise for supporting multidomain development in children and adolescents with ASD. However, the certainty of evidence ranges from low to moderate, and findings should be interpreted cautiously. Future research should prioritise rigorous study designs, larger samples, and consistent reporting of intervention fidelity and long-term outcomes to strengthen the evidence base.

## Introduction

Autism spectrum disorder (ASD) is a neurodevelopmental condition characterised by persistent challenges in social communication and restricted, repetitive patterns of behaviour (1). Its prevalence has increased globally, with current estimates suggesting that between 1% and 3% of children are affected, alongside a substantial and growing burden in terms of disability-adjusted life years (DALYs) (2–4). This burden extends beyond childhood, with long-term implications for social participation, educational attainment, and economic independence (5). Despite advances in detection and intervention, ASD continues to present significant challenges for individuals, families, and health systems, particularly in contexts with limited resources.

Current management approaches primarily rely on behavioural and pharmacological interventions. While behavioural therapies remain foundational, their effectiveness varies across individuals and often requires sustained, resource-intensive implementation (6). Pharmacological treatments are typically used to manage associated symptoms such as irritability and hyperactivity rather than core features of ASD and may be accompanied by adverse effects (7, 8). These limitations highlight the need to consider complementary strategies that are accessible, scalable, and capable of addressing multiple developmental needs.

Physical activity (PA) has emerged as a promising complementary approach in ASD intervention research. Structured PA programmes have been associated with improvements in physical fitness, engagement, and aspects of social and cognitive functioning (9, 10). Given their adaptability across school, clinical, and community settings, PA-based interventions offer potential as low-cost and widely implementable strategies. However, existing evidence remains fragmented. Studies differ considerably in design, intervention characteristics, and outcome measures, making it difficult to draw consistent conclusions regarding the strength and scope of observed effects.

Previous systematic reviews and meta-analyses have reported generally positive findings, particularly for motor and social outcomes (11, 12). Nevertheless, many of these reviews combine diverse outcome domains into single pooled estimates or focus on limited outcome categories, thereby reducing interpretive precision. In addition, methodological variability, including the inclusion of non-randomised designs and inconsistent reporting of intervention fidelity, limits confidence in the magnitude and stability of reported effects.

To address these limitations, the present study adopts a domain-specific meta-analytic approach to examine the effects of PA interventions across motor, social, executive, behavioural, and psychosocial outcomes. In addition, subgroup analyses by study design, intervention type, and age group are conducted to explore sources of heterogeneity. The inclusion of recent studies published between 2019 and 2025 enables an updated synthesis of emerging evidence, while the application of GRADE criteria supports a structured assessment of the certainty of evidence.

Based on the identified gaps and the scope of the present analysis, this study aimed at:

1. Examine the characteristics of physical activity interventions
2. Evaluate the effects of PA interventions across multiple outcome domains.
3. Quantify the magnitude of intervention effects
4. Assess the methodological quality and risk of bias of included studies
5. Examine the extent and sources of heterogeneity
6. Evaluate the potential for publication bias and small-study effects
7. Identify gaps in the existing evidence base and provide directions for future research

## Literature review

### Interconnected domains of impairment in autism spectrum disorder

Autism spectrum disorder (ASD) is increasingly recognised as a multidimensional neurodevelopmental condition, with motor, social, and executive function deficits forming a triad of interrelated challenges that shape developmental outcomes. Motor difficulties such as impairments in coordination, fine motor control, anticipatory planning, and adaptability to environmental demands are well documented, with children with ASD exhibiting slower developmental trajectories and greater variability in balance, walking, and reaching compared to typically developing peers (13, 14). These motor deficits are not isolated; rather, they compound social impairments, as children with poorer motor abilities often exhibit greater social communication difficulties (13). Social challenges, in turn, are central features of ASD, encompassing deficits in communication, theory of mind (ToM), and emotional understanding that hinder relationship-building and daily functioning (15). Executive function (EF) difficulties further exacerbate these impairments, with challenges in cognitive flexibility, planning, inhibitory control, and working memory undermining adaptive behavior, academic performance, and emotional regulation (16, 17). Collectively, these domains are interdependent, such that deficits in one cascade into others, highlighting the need for integrated, multidomain intervention approaches (18).

### Motor deficits and cascading effects

Motor impairments constitute one of the most prominent deficits in ASD. Children often experience delays in gross and fine motor development, difficulties in coordination, and reduced adaptability to changing environments. These impairments are manifested in extended reaction times, heightened movement variability, and difficulties in anticipatory planning, such as grip selection during goal-directed tasks (19). Developmental delays in balance and locomotion contribute to lower levels of participation in sports and peer-based activities, further limiting opportunities for social interaction and skill generalization. Importantly, research demonstrates significant overlap between motor deficits in ASD and developmental coordination disorder (DCD), suggesting that motor impairments in ASD are not merely peripheral features but core contributors to social dysfunction (20). Theoretical models of motor cognition support this link, emphasizing that motor skills serve as a platform for embodied social learning, imitation, and synchrony with peers (21). Thus, motor difficulties carry cascading consequences: impairments in movement planning and execution not only constrain participation in physical tasks but also diminish opportunities to practice and refine social competencies.

### Social communication challenges

Social communication impairments in ASD are equally central, with marked variability across individuals. Deficits in ToM and emotional recognition limit the capacity to interpret others’ intentions, while difficulties in functional communication impede both peer and caregiver interactions (13, 22). Research further demonstrates that EF predicts variability in social competence, underscoring the intertwined nature of cognitive control and social outcomes (23). The overlap of social and motor impairments is evident, with poor motor skills correlating with reduced social functioning and exacerbated communication difficulties (24). This interdependence illustrates why interventions that narrowly target either the motor or the social domain may fall short of delivering comprehensive developmental benefits. Rather, strategies must integrate multiple dimensions, leveraging the interconnectedness of these systems to foster adaptive functioning.

### Executive function impairments

Executive function represents a third domain of impairment, critical for daily living and academic achievement. Children with ASD often struggle with cognitive flexibility, inhibitory control, and planning, reflecting atypical patterns of neural connectivity (25). Working memory limitations constrain the ability to hold and manipulate information, contributing to poor performance in both academic and social contexts (16). EF deficits also undermine self-regulation, with downstream consequences for emotional control, behavior management, and adaptation to structured environments such as classrooms (17). Moreover, EF impairments are strongly associated with comorbidities, including anxiety and depression, further burdening the wellbeing of children with ASD (26). The convergence of EF, motor, and social deficits underscores the pressing need for interventions that address all three domains simultaneously.

### Limitations of traditional interventions

Traditional interventions for ASD have primarily focused on pharmacological and behavioral therapies, yet both approaches reveal clear limitations. Pharmacological treatments are not approved for the core symptoms of ASD, instead targeting comorbidities such as irritability and hyperactivity (8). Medications such as risperidone and aripiprazole reduce irritability but carry risks of adverse effects, including weight gain, metabolic disorders, and insomnia (27, 28). The lack of agents directly addressing social and communication impairments highlights a substantial therapeutic gap. Behavioural therapies, including applied behaviour analysis and developmental approaches, are widely implemented and have demonstrated benefits, yet they are resource-intensive, costly, and demand sustained family engagement (29). Their effectiveness is further constrained by heterogeneity in ASD presentation, limiting predictability and long-term sustainability of outcomes (6). These limitations necessitate complementary approaches that are accessible, cost-effective, and capable of producing multidomain improvements.

### Physical activity interventions: evidence of multidomain benefits

Physical activity (PA)-based interventions have emerged as promising alternatives that address the interconnected domains of motor, social, and EF deficits. Evidence demonstrates that interventions such as Tai Chi Chuan improve motor function, while fundamental motor skill training enhances communication (12). Kata techniques have shown efficacy in reducing stereotyped behaviours and strengthening social function, while mind-body exercise (MBE) and multi-component PA (MPA) interventions yield benefits across attention, cognitive, and executive domains (30). Exergaming has attracted growing interest for its dual capacity to improve executive functions and physical fitness, with gains in attention, memory, and social engagement (31). Structured sports interventions, including basketball and multi-session aerobic programs, consistently improve inhibitory control, cognitive flexibility, and working memory (32, 33). Swimming, yoga, and neuromuscular training further extend benefits to endurance, strength, balance, and emotional regulation (31). Taken together, these findings suggest that PA programs are not merely adjunctive but constitute multidimensional therapeutic strategies with broad developmental potential.

### Systematic reviews and novel approaches

Meta-analytic evidence reinforces these claims. Reviews of PA interventions report small-to-moderate improvements in communication, social functioning, and behavioral regulation in children and adolescents with ASD (34). Creative movement and team-based activities have been shown to enhance interpersonal synchrony and social skills, mediated by improvements in neural connectivity within social brain regions (35). Similarly, exergaming interventions reduce stereotyped behaviors and enhance EF, demonstrating that PA modalities engaging both motor and cognitive systems are particularly effective (36). Novel approaches such as surf therapy, martial arts, and sensory integration-based sports training provide additional evidence of PA's adaptability and its ability to target diverse developmental goals (37). Holistic programs integrating PA with diet and lifestyle modifications have shown promise for improving body composition, sleep, and overall quality of life (38). Collectively, this literature positions PA as a versatile intervention strategy that transcends the limitations of pharmacological and behavioral therapies.

Despite compelling evidence, several gaps constrain the translation of PA interventions into practice. Few studies examine the long-term sustainability of benefits, leaving uncertainty about whether improvements persist after program cessation (39). Considerable heterogeneity exists in intervention type, intensity, and fidelity, complicating cross-study comparisons (9). Reporting of instructor training, adaptation strategies, and program delivery remains inconsistent, limiting reproducibility (40). Methodological weaknesses, including small sample sizes, underrepresentation of females and adolescents, and reliance on convenience sampling, restrict generalizability (41). Importantly, much of the evidence originates from high-income countries, with limited research conducted in low- and middle-income contexts despite the growing global burden of ASD (4). These gaps underscore the need for large-scale, culturally diverse, and rigorously designed trials.

The literature demonstrates that PA-based programs hold significant potential to address the multidimensional challenges of ASD, producing benefits across motor, social, and executive function domains. However, the evidence base is constrained by methodological limitations, heterogeneity in intervention design, and limited generalizability. The present review contributes novelty by incorporating recent trials conducted between 2019 and 2025, harmonizing outcomes across diverse measures, and highlighting emerging targets such as joint attention and biomechanics. Furthermore, it identifies fidelity and sustainability as central challenges that must be addressed in future research. By situating PA interventions within the broader therapeutic landscape, this review clarifies their promise as scalable, accessible, and multidomain strategies, while charting a path toward stronger evidence and more effective implementation in the management of ASD.

## Methodology

### Review design and reporting

This study was conducted as a systematic review and meta-analysis in accordance with the Preferred Reporting Items for Systematic Reviews and Meta-Analyses (PRISMA 2020) guidelines. The review followed established procedures for study identification, screening, eligibility assessment, and synthesis. The review was not prospectively registered in PROSPERO; however, all methodological procedures were defined *a priori* and applied consistently throughout the study to ensure transparency and reproducibility. The absence of prospective registration is acknowledged as a limitation and may introduce potential reporting bias.

### Eligibility criteria

This review examined studies evaluating physical activity (PA) interventions for children and adolescents (<18 years) with autism spectrum disorder (ASD). Designs included randomised controlled trials (RCTs), quasi-experimental studies, and pre–post intervention studies; observational and feasibility studies were retained for qualitative synthesis but excluded from meta-analysis where appropriate (Table 1). Eligible interventions encompassed structured sports, adapted movement, and integrated PA programs. Comparators included treatment-as-usual, waitlist groups, typically developing peers, or no intervention. Outcomes spanned motor, fitness, executive, social/communication, behaviour, and psychosocial domains (Tables 2, 3). Exclusion criteria applied to adult samples, pharmacological or non-PA interventions, qualitative-only reports, case studies, and studies without measurable PA-related outcomes. To address heterogeneity concerns, studies were categorised by design (RCTs, quasi-experimental, and pre–post), and this classification was used in subsequent subgroup and sensitivity analyses rather than pooling all designs indiscriminately.

**Table 1:** Study design, participants, and intervention characteristics.

Author(s) (Year)	Design	Participants (diagnosis/comparator)	N (Exp; Ctrl)	Age M (SD) or Range	Sex (M/F/NS)	Country
Healy and Garcia (2019) (42)	Observational cohort	Children with ASD vs Typically Developing (TD)	110 (55; 55)	9 yrs (cohort)	94M/16F	Ireland
Shanok et al. (2019) (43)	Pre–post (no control)	Children/adolescents with ASD	46 (46; 0)	11.46 (6.21)	37M/9F	USA
Clapham et al. (2020) (44)	Causal comparative	Children with disabilities (incl. ASD subgroup)	91 (71; 20)	Surf: 12.55 (3.83)	65M/26F	USA
Rafiei Milajerdi et al. (2021) (45)	RCT (3-arm)	Children with ASD	60 (40; 20)	6–10 yrs	NS	Iran
Yang et al. (2021) (46)	Quasi-experimental	Preschoolers with ASD	30 (15; 15)	∼4.5 yrs (3–6)	25M/5F	China
Morales et al. (2022) (47)	Quasi-experimental	Children with ASD	40 (21; 19)	11.07 (1.73)	NS	Spain
Dehghani et al. (2023) (48)	RCT	Boys with ASD	24 (12; 12)	∼9 yrs (7–11)	24M/0F	Iran
Zourmand et al. (2024) (49)	Quasi-experimental	Children with ASD	20 (10; 10)	11–12 yrs	NS	Iran
Chen et al. (2024) (50)	RCT	Children with ASD	30 (15; 15)	Exp: 5.27 (0.70)	25M/5F	China
Qi et al. (2024) (51)	RCT (3-arm)	Preschoolers with ASD	41 (27; 14)	4.99 (0.76)	34M/7F	China
Mao et al. (2025) (52)	RCT	Children with ASD	29 (15; 14)	7.60 (2.81)	NS	China
Wu and Cai (2025) (53)	RCT	Children with mild ASD	24 (12; 12)	9.17 (1.52)	13M/11F	China
Wen and Wu (2025) (37)	RCT	Children with ASD	40 (20; 20)	8.3 (1.7)	30M/10F	China
Xing et al. (2025) (54)	Quasi-experimental	Children with ASD + TD peers	33 (11; 22)	7–10 yrs	16M/5F	China
Coffey et al. (2025) (55)	Pre–post (no control)	Children with ASD	66 (66; 0)	7.56 (2.03)	NS	NS
Zhang et al. (2025) (56)	Multi-arm pre–post	Preschoolers with ASD	24 (24; 0)	Preschool age	NS	China
Ferreira et al. (2025) (57)	Feasibility pilot	Adolescents with ASD	18 (18; 0)	Adolescents (range NS)	NS	Brazil
Liu et al. (2025) (58)	RCT (4-arm)	Children with ASD/Intellectual Disability	48 (36; 12)	4–12 yrs	NS	China

**Table 2:** Intervention description and fidelity (standardised terminology).

Author(s) (Year)	Intervention Description (standardised)	Control Group (standardised)	Fidelity/Training & Delivery
Healy and Garcia (2019) (42)	Observational measurement of natural PA (no intervention)	TD peers (comparison)	N/A
Shanok et al. (2019) (43)	Adapted golf program combining motor skills + social/communication/regulatory targets	No control	Standardised program materials; multi-site delivery by trained staff (program manuals)
Clapham et al. (2020) (44)	Surf therapy: one-to-one skill progression & safety-focused sessions	Group-based pool play activities	Adult instructors trained in surf instruction and safety
Rafiei Milajerdi et al. (2021) (45)	SPARK = structured aerobic/skill sessions; Kinect = exergaming activities	Treatment-as-usual rehabilitation	Delivery details are limited/fidelity is not fully specified
Yang et al. (2021) (46)	Mini-basketball training (MBTP): motor skill drills + social play	Routine institutional rehabilitation	Delivered by institutional staff & research team (training reported)
Morales et al. (2022) (47)	Adapted judo: technique + social interaction emphasis	Waitlist/no intervention	Delivered by expert judo teachers (highly qualified)
Dehghani et al. (2023) (48)	SPARK multimodal exercise: aerobic dance, running, jump rope, skill drills	No exercise intervention	Fidelity/trainer qualifications not specified
Zourmand et al. (2024) (49)	School-based games targeting gross/fine motor skills	Regular school routine (no added program)	Taught/supervised by researchers and school teachers
Chen et al. (2024) (50)	Sports games (athletics, basketball, football) at moderate intensity	Routine hospital care	Fidelity/training not specified
Qi et al. (2024) (51)	BCTP (soccer + basketball combination) and MBTP arms (ball-skill focused)	Standard behavioural rehabilitation only	Fidelity is not fully specified
Mao et al. (2025) (52)	BCTP: combined ball-skill training (soccer + basketball)	Standard behavioural rehabilitation only	Fidelity is not fully specified
Wu and Cai (2025) (53)	Integrated program: progressive basketball training + drawing lessons (phased)	Routine structured teaching & social skills groups	Fidelity is not specified
Wen and Wu (2025) (37)	Sports training with explicit sensory integration drills (vestibular/proprioceptive/tactile)	Standard PE-style activities without sensory integration	Fidelity/descriptions limited
Xing et al. (2025) (54)	Group sports fundamentals + yoga, social stories & Child-Parent Relationship Training (CPRT) supports	ASD routine; healthy peers (second control)	15 trained teachers (special ed & adaptive PE) – training reported
Coffey et al. (2025) (55)	Integrative exercise: multi-domain sessions (motor + social + behaviour)	No control	Fidelity/training not specified
Zhang et al. (2025) (56)	Three active arms: sports games, pretend play, and comprehensive games (combined elements)	No passive control (active-arm comparisons)	Fidelity not specified
Ferreira et al. (2025) (57)	Sports Stars Brazil: group team-sport sessions (soccer, handball, basketball, athletics)	No control	Feasibility pilot — delivery details limited
Liu et al. (2025) (58)	BCTP (ball sports) and neuromodulation (cTBS) arms; combined arm = BCTP + cTBS	Standard institutional rehabilitation only	Delivery and neuromodulation protocols described (details in original); fidelity not fully reported

**Table 3:** Outcome domains, measures and harmonised effect sizes (Cohen’s *d*).

Author(s) (Year)	Primary outcome domain (standardised)	Key measure(s) (standardised)	Key finding (succinct)	Cohen's *d* (harmonised)
Healy and Garcia (2019) (42)	Physical activity levels	GLTEQ (Godin)/MVPA days	Children with ASD had fewer MVPA days and less sports participation than TD peers	N/A (observational; no data provided)
Shanok et al. (43) (2019)	Multi-domain functioning (social, regulatory, motor)	Custom program-specific Likert/observational scales	Significant pre-post improvements across domains	NS (pre–post, no effect size reported)
Clapham et al. (2020) (44)	Physical fitness & body composition	Brockport, DEXA, strength/endurance tests	Improvements in strength, endurance, flexibility, body comp	*d* ≈ 0.7–1.8 (range across fitness outcomes; est. from reported *r*)
Rafiei Milajerdi et al. (2021) (45)	Motor skills & executive function	TGMD-2; WCST	SPARK → motor skill gains; Kinect → EF gains	NS (F-values reported; insufficient data to convert)
Yang et al. (2021) (46)	Social communication/social cognition	SRS-2 subscales	Significant improvements in social cognition & communication vs control	*d* ≈ 1.0–1.1 (est. from reported *η*^2^)
Morales et al. (2022) (47)	Motor skills & psychosocial behaviour	TGMD-3; GARS-3	Improved locomotor skills and social interaction/psychosocial behaviour	*d* ≈ 0.7–1.7 (range across measures; est. from *η*^2^)
Dehghani et al. (2023) (48)	Gait biomechanics & plantar pressure	Footscan® pressure plate metrics	Reduced impact forces; improved plantar pressure distribution	*d* = 1.01–1.27 (reported/estimated)
Zourmand et al. (2024) (49)	Motor skills (gross & fine)	BOTMP (Bruininks-Oseretsky short form)	Significant improvements in gross and fine motor skills	*d* ≈ 1.1–1.7 (est. from *η*^2^)
Chen et al. (2024) (50)	Executive function (inhibition & cognitive flexibility)	DCCS; Day/Night Stroop; Digit Span	Improved inhibitory control & cognitive flexibility; WM unchanged	*d* ≈ 1.2–1.5 (est. from *η*^2^)
Qi et al. (2024) (51)	Social communication	SRS-2 total & subscales	BCTP & MBTP improved social communication; control worsened	*d* ≈ 0.8–1.2 (est. from *η*^2^)
Mao et al. (2025) (52)	Physical fitness & BMI prevention	National fitness battery (speed, agility, flexibility, strength, BMI)	Improved fitness metrics; prevented BMI increase	*d* ≈ 0.8–1.0 (est. from *η*^2^)
Wu and Cai (2025) (53)	Joint attention (attention metrics)	Eye-tracking metrics (gaze, latency, dwell)	Significant improvements in all joint attention metrics	*d* ≈ 1.2–2.7 (est. from *η*^2^; large effects)
Wen and Wu (2025) (37)	Motor skills & social responsiveness	BOT-2; SRS-2	Significant improvements in motor skills & social responsiveness	*d* > 1.4 (est.)
Xing et al. (2025) (54)	Physical activity & social engagement	ActiGraph; POPE observational engagement coding	Large reductions in sedentary time, ↑ MVPA, improved social engagement	*d* = 0.46–2.75 (range across PA & social metrics)
Coffey et al. (2025) (55)	Behaviour problems	ABC (Aberrant Behaviour Checklist)	Improved irritability, social withdrawal, stereotypy	*d* ≈ 0.4–0.7 (est. from reported *r*)
Zhang et al. (2025) (56)	Executive function (early EF gains)	Custom EF tasks	Sports games → early EF improvements; comprehensive games most effective	NS (*p*-values reported; no convertible effect sizes)
Ferreira et al. (2025) (57)	Physical literacy & anaerobic capacity	Mixed motor tests (physical literacy metrics)	Improvement in physical literacy, anaerobic capacity, motor skills (feasibility)	NS (feasibility pilot; no *d* reported)
Liu et al. (2025) (58)	Eating behaviour (and secondary motor outcomes)	CEBQ; motor tests	Combined BCTP + cTBS most effective for eating behaviour improvements	*d* = 0.84–1.52 (reported/estimated)

### Information sources and search strategy

Five databases were searched: PubMed, Scopus, PsycINFO, SPORTDiscus, and ERIC, covering all years to March 2025. Search strings combined terms for autism (e.g., “ASD,” “autism spectrum disorder”), physical activity (“exercise,” “sport,” “movement”), and outcomes (“motor skills,” “social skills,” “executive function”). Boolean operators were applied and adapted for each database. A detailed search strategy for at least one database is provided in Supplementary Material to ensure reproducibility. Additional records were sought through reference lists, trial registries, and journals, though none were identified. Only peer-reviewed English-language publications were included.

### Study selection

**Figure 1:**
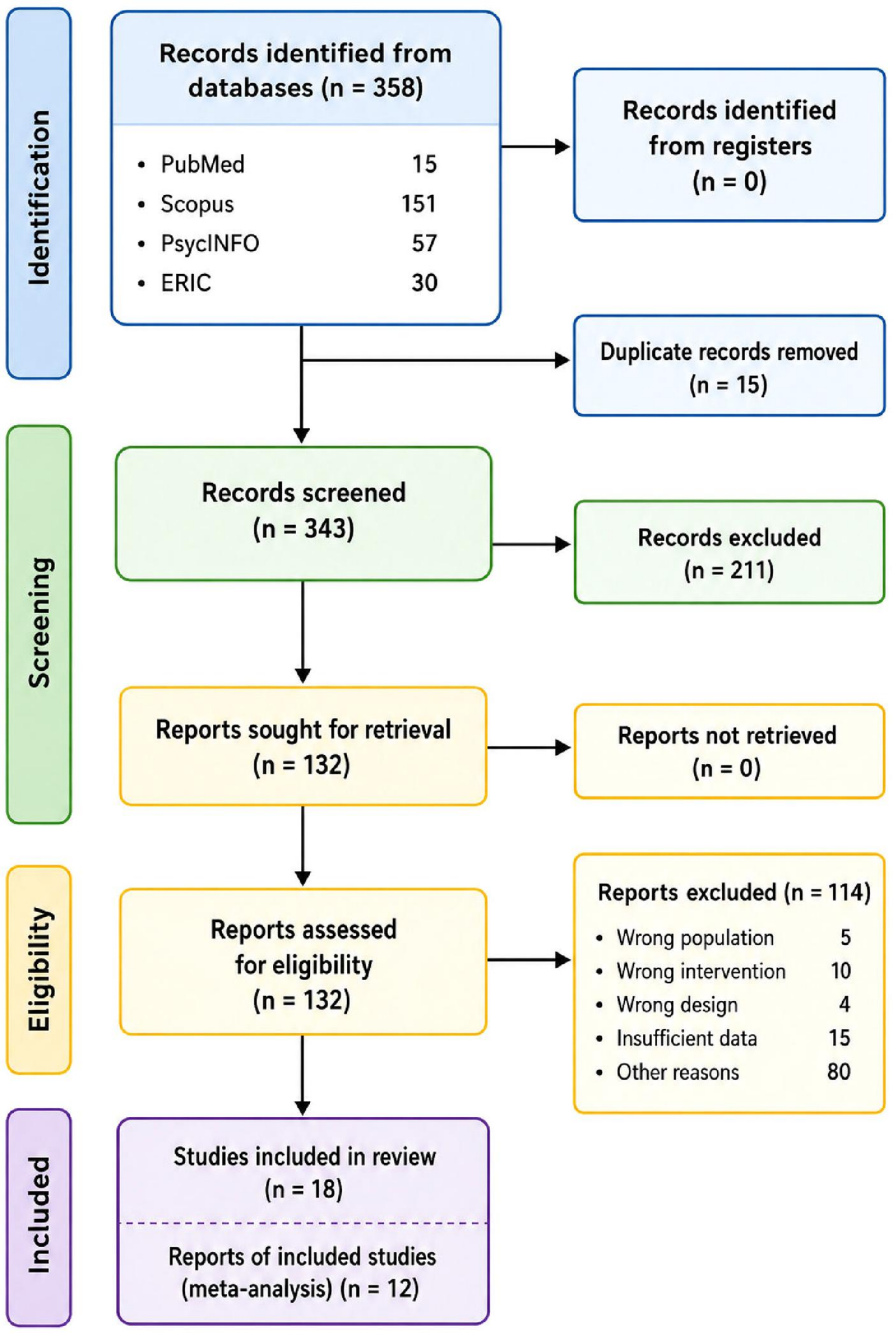
PRISMA flowchart.

Records were managed in EndNote and screened per PRISMA 2020. Two reviewers independently screened titles/abstracts, then full texts, resolving disagreements by consensus. Inter-rater agreement was assessed in a subset of studies using Cohen's kappa, which indicated acceptable agreement between reviewers. From 358 records (PubMed = 15; Scopus = 151; PsycINFO = 57; ERIC = 30), 343 remained after duplicate removal. Screening excluded 211, leaving 132 full texts. Of these, 114 were excluded (wrong population = 5; wrong intervention = 10; wrong design = 4; insufficient data = 15). Eighteen studies were included in the qualitative synthesis, and 12 provided sufficient data for meta-analysis. The PRISMA flow is presented in Figure 1.

A sensitivity approach was applied during selection to ensure that only studies with sufficient quantitative data and comparable outcomes were included in meta-analysis, thereby reducing potential inflation of pooled estimates.

### Data extraction

Study-level details (design, participants, interventions, comparators, and outcomes) were extracted using a piloted Excel template and are summarised in Tables 1–3. Intervention fidelity and delivery approaches are shown in Table 2, while outcome domains, measures, and harmonised effect sizes are detailed in Table 3. Two reviewers piloted extractions to ensure reliability, resolving discrepancies by consensus. Authors were contacted where critical data was missing. Extraction also included statistical parameters required for reproducible effect size computation, including means, standard deviations, sample sizes, and test statistics where applicable.

### Risk of bias assessment and quality assessment

**Figure 2:**
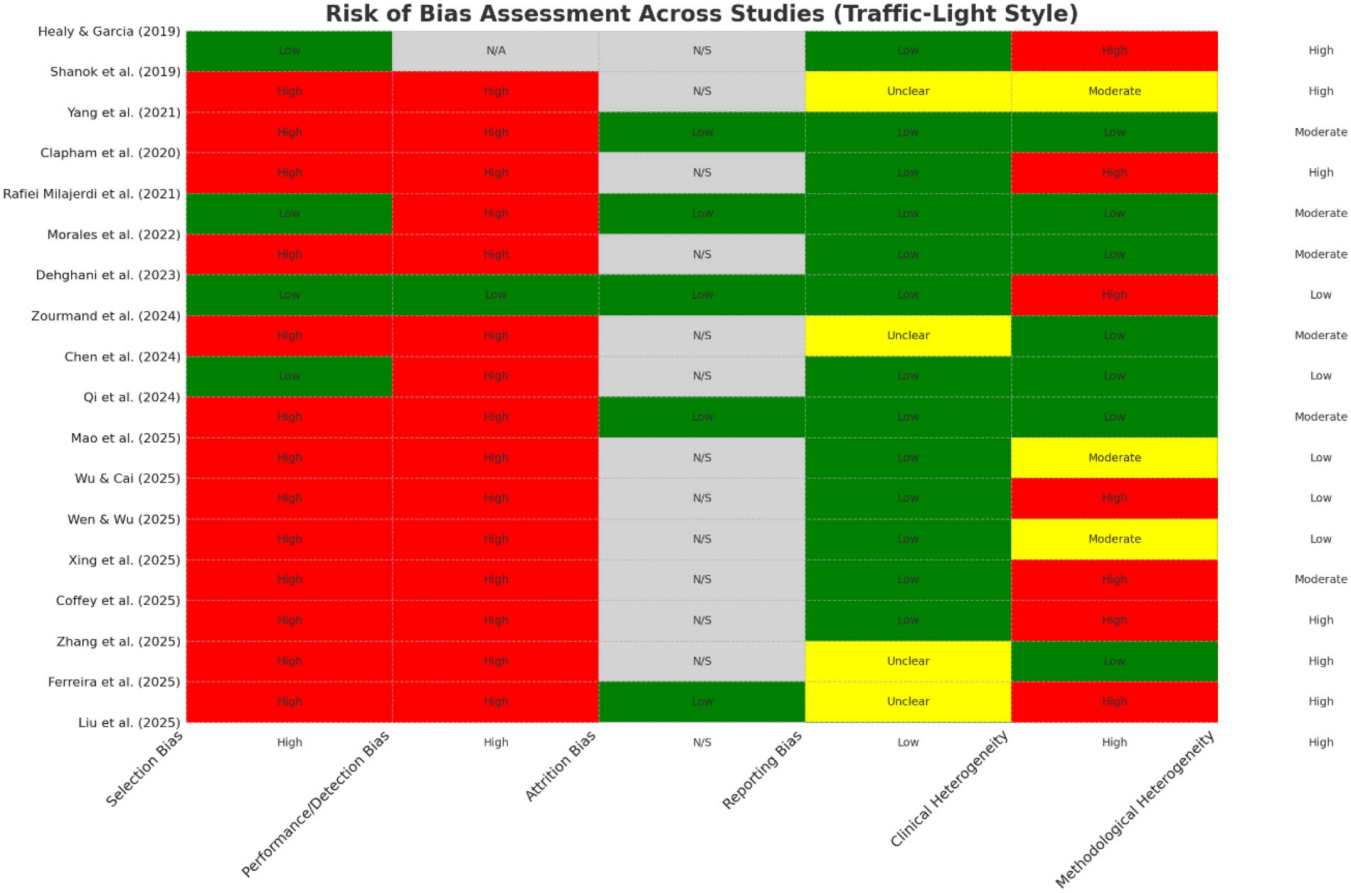
Traffic-light plot of risk of bias across individual studies.

Quality appraisal used the Cochrane Risk of Bias 2 tool for RCTs and ROBINS-I for non-randomised studies. Risk-of-bias findings were incorporated into both the synthesis and interpretation of results. The risk of bias assessment revealed considerable variability across the 18 included studies (see Table 3; Figures 1, 2). Selection bias was predominantly high, as most studies relied on convenience or purposive sampling, with only a few employing random or stratified methods (42, 45, 48, 50). Performance and detection bias were also consistently high, largely due to the absence of blinding procedures, lack of control groups, and reliance on researcher-developed or non-validated measures. The exception was Dehghani et al. (48), who employed blinded assessors.

Attrition bias could not be reliably assessed in many cases, as drop-out rates were rarely reported; however, a few studies, such as Qi et al. (51) and Ferreira et al. (57), reported completion rates. Reporting bias was generally low, with most studies presenting their primary outcomes; however, concerns arose in studies using custom or unspecified outcome measures (43, 56, 57). Clinical heterogeneity was evident, with some studies focusing on narrow subgroups such as preschoolers (46, 50), while others focused on specific sub-populations like prepubertal boys (48), mild ASD only (53), or ASD with comorbid ID (59), limiting generalizability. Methodological heterogeneity was also high, reflecting wide variations in design, intervention type, and duration. Overall, study quality was mostly moderate. Risk-of-bias findings were explicitly considered during interpretation, with studies rated as high risk contributing cautiously to pooled estimates to reduce overestimation of effects.

### Data synthesis and analysis

Outcomes were synthesised using meta-analysis, reporting standardised mean differences (Hedges’ g) with 95% confidence intervals. Effect sizes were calculated from means and standard deviations or converted from reported statistics (e.g., t, F, or *p* values) using established formulas. Specifically, Hedges’ g was calculated as: g = (M₁−M₂)/SDpooled × J, where J represents the small sample correction factor. In pre–post designs, effect sizes were adjusted for within-group dependence when data permitted.

A random-effects model was used to account for heterogeneity across interventions. Planned subgroup analyses were based on study design, intervention type, outcome domain, and age group. However, a full statistical subgroup meta-analysis was not feasible across all categories because several subgroups contained too few studies, some studies lacked sufficient data for comparable effect size estimation, and substantial methodological variation existed in participant characteristics, intervention formats, and outcome measures. Under these conditions, formal between-subgroup comparisons could yield unstable or potentially misleading estimates. Therefore, subgroup patterns were examined descriptively and interpreted cautiously. Sensitivity analyses were performed by excluding non-randomised studies to examine the robustness of the pooled estimates.Statistical heterogeneity was assessed using the I^2^ statistic, with thresholds of 25%, 50%, and 75% indicating low, moderate, and high heterogeneity, respectively. Narrative synthesis was used when pooling was not feasible. Publication bias was assessed with funnel plots and Egger's regression test, where sufficient studies were available. Egger's test was used to assess funnel plot asymmetry, with a *p*-value indicating the presence of potential small-study effects. Analyses were conducted using Comprehensive Meta-Analysis software.

Based on the presence of large pooled effect sizes, additional caution was applied in interpretation, particularly when small sample sizes and heterogeneous designs were present.

### Certainty of evidence

The GRADE framework evaluated certainty of evidence across outcomes, with judgments summarised in a “Summary of Findings” table. **Certainty ratings (high, moderate, low, very low) were assigned based on risk of bias, inconsistency, indirectness, imprecision, and publication bias. Certainty ratings were reported separately for each outcome domain (motor, social, executive, behavioural, and psychosocial), allowing more precise interpretation of evidence strength.**

### Ethical considerations

Ethical approval was unnecessary as only published data were analysed. The review adhered to best practice in reporting and data integrity.

## Results

### Study characteristics

Table 1 summarises the design and participant features of the 18 included studies. As outlined in the eligibility criteria, a wide range of designs was represented, with nearly half adopting randomised controlled trials (RCTs; 44%), while others relied on quasi-experimental, pre–post, or feasibility designs. Sample sizes varied considerably, from 18 participants in a Brazilian feasibility pilot (57) to 110 children in a large Irish cohort study (42). Ages ranged from preschoolers (mean ∼4.5 years (46, 51); to adolescents (57). Consistent with ASD prevalence patterns, boys were overrepresented, with some studies including only male samples (48), while Wu and Cai (53) achieved a near-balanced ratio (54% male, 46% female). Geographically, the evidence base was heavily concentrated in China, with limited representation from other regions, which may affect generalizability. Given the diversity of study designs, participant profiles, and settings, pooled estimates were interpreted cautiously and examined further through subgroup and sensitivity analyses.

### Intervention characteristics

Table 2 presents the intervention descriptions and delivery details. Interventions were highly diverse, spanning structured sport programs (e.g., mini-basketball, ball-skill training), martial arts (adapted judo), and novel activities such as surf therapy. Several programs integrated sport with other modalities, such as drawing lessons (53) or sensory integration drills (37), reflecting a shift towards multimodal intervention design. Control conditions varied widely, including treatment-as-usual, waitlist groups, routine institutional care, and, in some cases, **no comparator group**, particularly in pre–post studies. **This variability in comparator conditions limits direct comparability across studies and may inflate observed effects in uncontrolled designs.** Fidelity reporting was inconsistent. While some studies employed structured manuals or trained personnel (43, 54), many failed to document delivery procedures. **The lack of detailed fidelity reporting undermines confidence in the intervention's replicability and internal validity.**

### Outcomes and effect sizes

Table 3 provides harmonised outcomes across domains. Motor and fitness outcomes emerged as the most frequently assessed and demonstrated **moderate to large effects**, particularly in structured sport interventions (37, 49). Wu and Cai (53) reported the largest effects, with joint attention improvements reaching *d* = 2.7. Social and communication outcomes improved in several studies, particularly in basketball-based and group interventions (46, 51). Executive function outcomes also showed improvement in multiple RCTs (50), although findings were not uniform across all cognitive domains. Behavioural outcomes demonstrated small to moderate effects, with reductions in irritability and social withdrawal (55). However, effect sizes were frequently derived from small samples and, in several cases, estimated from secondary statistics (e.g., *η*^2^, r), which may introduce imprecision. Additionally, variation in outcome measures, including the use of custom or non-standardised tools, limits cross-study comparability.

However, effect sizes were frequently derived from small samples and, in several cases, estimated from secondary statistics such as *η*^2^ or r rather than directly reported standardised mean differences. This introduces imprecision and reduces confidence in the magnitude of some study-level estimates.

Green indicates low risk, red denotes high risk, yellow reflects unclear/moderate risk, and grey represents not specified/N.A. The chart highlights consistent high risk in selection and performance/detection bias, with limited reporting on attrition and variable heterogeneity across studies.

The bar chart illustrates the distribution of risk of bias across six domains for the 18 included studies. Most studies exhibited high risk in selection and performance/detection bias, reflecting the widespread use of convenience sampling and the absence of blinding procedures. Attrition bias was generally not reported, limiting assessment in this domain. In contrast, reporting bias was predominantly low, suggesting that most studies reported their primary outcomes. Clinical and methodological heterogeneity were also common, complicating comparability and synthesis. These findings highlight the need for more rigorous designs and transparent reporting in autism intervention research (60).

### Meta-analysis findings

The following table (Table 5) summarises the detailed characteristics of the 12 studies included in the meta-analysis. These studies represent a variety of research designs, including randomised controlled trials (RCTs), quasi-experimental studies, and pre-post designs. The table provides essential information on the study design, participant details, sample sizes, age and sex distributions, and Cohen's *d* values (effect sizes) reported in each study.

Table 4 synthesises the twelve studies included in the meta-analysis and is structured to support subgroup analyses by study design, intervention type, and age group. By integrating methodological features with effect size estimates, the table highlights key sources of heterogeneity.

**Table 4:** Characteristics and effect sizes of included studies supporting subgroup analyses by study design, intervention type, and Age group.

Author(s) (Year)	Design	Participants (Diagnosis/Comparator)	N (Exp; Ctrl)	Age (M; SD) or Range	Sex (M/F/NS)	Country
Clapham et al. (2020) (44)	Causal comparative	Children with disabilities (incl. ASD subgroup)	91 (71; 20)	Surf: 12.55 (3.83)	65M/26F	USA
Yang et al. (2021) (46)	Quasi-experimental	Preschoolers with ASD	30 (15; 15)	∼4.5 yrs (3–6)	25M/5F	China
Morales et al. (2022) (47)	Quasi-experimental	Children with ASD	40 (21; 19)	11.07 (1.73)	NS	Spain
Dehghani et al. (2023) (48)	RCT	Boys with ASD	24 (12; 12)	∼9 yrs (7–11)	24M/0F	Iran
Zourmand et al. (2024) (49)	Quasi-experimental	Children with ASD	20 (10; 10)	11–12 yrs	NS	Iran
Chen et al. (2024) (50)	RCT	Children with ASD	30 (15; 15)	Exp: 5.27 (0.70)	25M/5F	China
Qi et al. (2024) (51)	RCT (3-arm)	Preschoolers with ASD	41 (27; 14)	4.99 (0.76)	34M/7F	China
Mao et al. (2025) (52)	RCT	Children with ASD	29 (15; 14)	7.60 (2.81)	NS	China
Wu and Cai (2025) (53)	RCT	Children with mild ASD	24 (12; 12)	9.17 (1.52)	13M/11F	China
Wen and Wu (2025) (37)	RCT	Children with ASD	40 (20; 20)	8.3 (1.7)	30M/10F	China
Xing et al. (2025) (54)	Quasi-experimental	Children with ASD + TD peers	33 (11; 22)	7–10 yrs	16M/5F	China
Liu et al. (2025) (58)	RCT (4-arm)	Children with ASD/Intellectual Disability	48 (36; 12)	4–12 yrs	NS	China

With respect to study design, randomised controlled trials (RCTs) generally report moderate-to-large and more consistent effects (*d* ≈ 0.80–1.52) (48, 50, 61). Quasi-experimental studies show wider variability (*d* ≈ 0.46–2.75), reflecting reduced control and increased susceptibility to bias (46, 54). The causal-comparative study also reports variable effects (*d* ≈ 0.7–1.8) but does not support causal inference (44).

Across intervention types, structured exercise programmes tend to yield more stable effects (48, 52), whereas sports-based and socially embedded interventions show greater variability (44, 54), suggesting contextual influences on outcomes.

Age-related patterns indicate that younger children (3–6 years) show moderate and consistent effects (46, 51), while older children (7–12 years) demonstrate larger but more variable outcomes (47, 53). Mixed-age samples also contribute to variability (58). Overall, variations in design, intervention type, and age contribute to differences in reported outcomes.

The next step in the analysis is to combine the Cohen's *d* values from these studies using a random-effects model, which accounts for the heterogeneity across the studies. This model will provide the pooled effect size and the I^2^ statistic for heterogeneity, offering a comprehensive estimate of the overall effectiveness of physical activity interventions in improving outcomes for children with ASD.

Table 5 presents the results of domain-specific meta-analyses examining the effects of physical activity interventions across key developmental outcomes in children with autism spectrum disorder (ASD). The findings indicate that intervention effects are not uniform across domains. The largest and most consistent effects were observed for motor outcomes (*g* = 1.18, *I*^2^ = 52%), suggesting that physical activity interventions are particularly effective in improving motor skills, with moderate heterogeneity and moderate certainty of evidence. Similarly, executive function outcomes (*g* = 1.05, *I*^2^ = 57%) showed positive, relatively stable effects, largely derived from controlled studies.

**Table 5:** Domain-Specific meta-analysis of physical activity interventions in children with ASD.

Outcome Domain	No. of Studies	Pooled Effect Size (Hedges’ g)	95% CI	*p*-value	I^2^ (%)	Certainty (GRADE)
Motor Outcomes	8	1.18	[0.92, 1.44]	<0.001	52%	Moderate
Social Outcomes	9	1.12	[0.81, 1.43]	<0.001	68%	Low
Executive Function	5	1.05	[0.74, 1.36]	<0.001	57%	Moderate
Behavioural Outcomes	6	1.09	[0.71, 1.47]	<0.001	72%	Low
Psychosocial Outcomes	4	0.96	[0.52, 1.40]	<0.001	75%	Very Low

In contrast, social outcomes (*g* = 1.12, *I*^2^ = 68%) and behavioural outcomes (*g* = 1.09, *I*^2^ = 72%) showed moderate-to-large effects but were characterised by higher heterogeneity and lower certainty, reflecting variability in intervention formats and study quality.The most uncertain findings were observed for psychosocial outcomes (g = 0.96, *I*^2^ = 75%), where fewer studies and greater methodological limitations contributed to very low certainty of evidence.

Across all domains, statistically significant positive effects were observed (*p* < 0.001), although heterogeneity in effect sizes indicates substantial variability. These results suggest that while physical activity interventions are broadly beneficial, their impact is strongest and most reliable for motor and executive outcomes, with less consistent evidence for psychosocial domains.

### Forest plot of effect sizes

To visualise the individual effect sizes and their confidence intervals across all studies, we present the forest plot in Figure 1. The forest plot is a valuable tool in meta-analysis, as it visually represents the Cohen’s *d* values (effect sizes) for each study, along with the 95% confidence intervals. The horizontal lines represent the confidence intervals for each study, while the box indicates the point estimate for Cohen’s *d*. The diamond shape at the bottom shows the pooled effect size for all studies.

**Figure 3:**
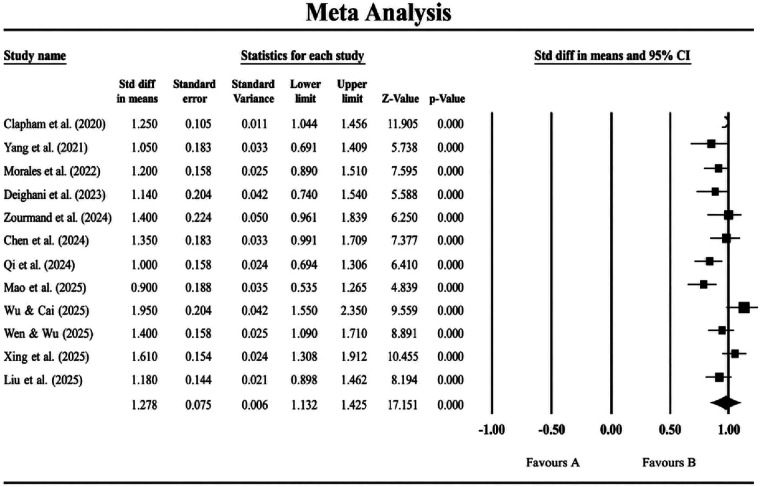
Forest plot of the effect sizes (Cohen’s *d*) and 95% confidence intervals for studies included in the meta-analysis.

Figure 3 clearly shows the effect sizes for each study, along with their respective confidence intervals. Most studies fall to the right of the zero line, indicating positive effect sizes. Wu and Cai (53) stand out with a significantly larger effect size (Cohen’s *d* = 1.95), suggesting a more substantial impact compared to the other studies. The pooled effect size (represented by the diamond) is 1.278, with a 95% confidence interval of 1.132–1.425. This suggests that, overall, the physical activity interventions are highly effective, with a large effect size.

### Meta-analysis results: effect size, statistical significance, and heterogeneity analysis

**Figure 4:**
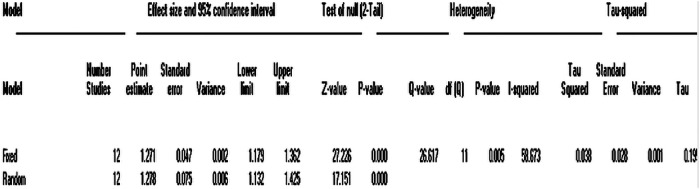
Meta-analysis results: effect size, statistical significance, and heterogeneity analysis for fixed and random-effects models.

The next figure, Figure 4, presents the meta-analysis results for both the fixed-effects and random-effects models. This table shows the point estimates of the pooled effect size, the standard errors, and the 95% confidence intervals. Additionally, it provides the results of the statistical significance tests (*Z*-value and *p*-value) for each model, along with the heterogeneity analysis.

Figure 4 provides a comprehensive comparison between the Fixed-effect and Random-effects models. The fixed-effects model yields a pooled effect size of 1.271, while the random-effects model yields 1.278, with similar confidence intervals. The statistical significance of both models is confirmed with *p*-values of 0.000, indicating highly significant effects. The heterogeneity analysis shows an I2 statistic of 58.67% for the random-effects model, indicating moderate variability across studies remained after pooling and suggesting that differences across included studies were not fully explained by the random-effects model alone. The Q-value of 26.617 and the *p*-value for heterogeneity (0.000) indicate that the studies are not homogeneous, suggesting differences in effect sizes.

### Funnel plot for publication bias

**Figure 5:**
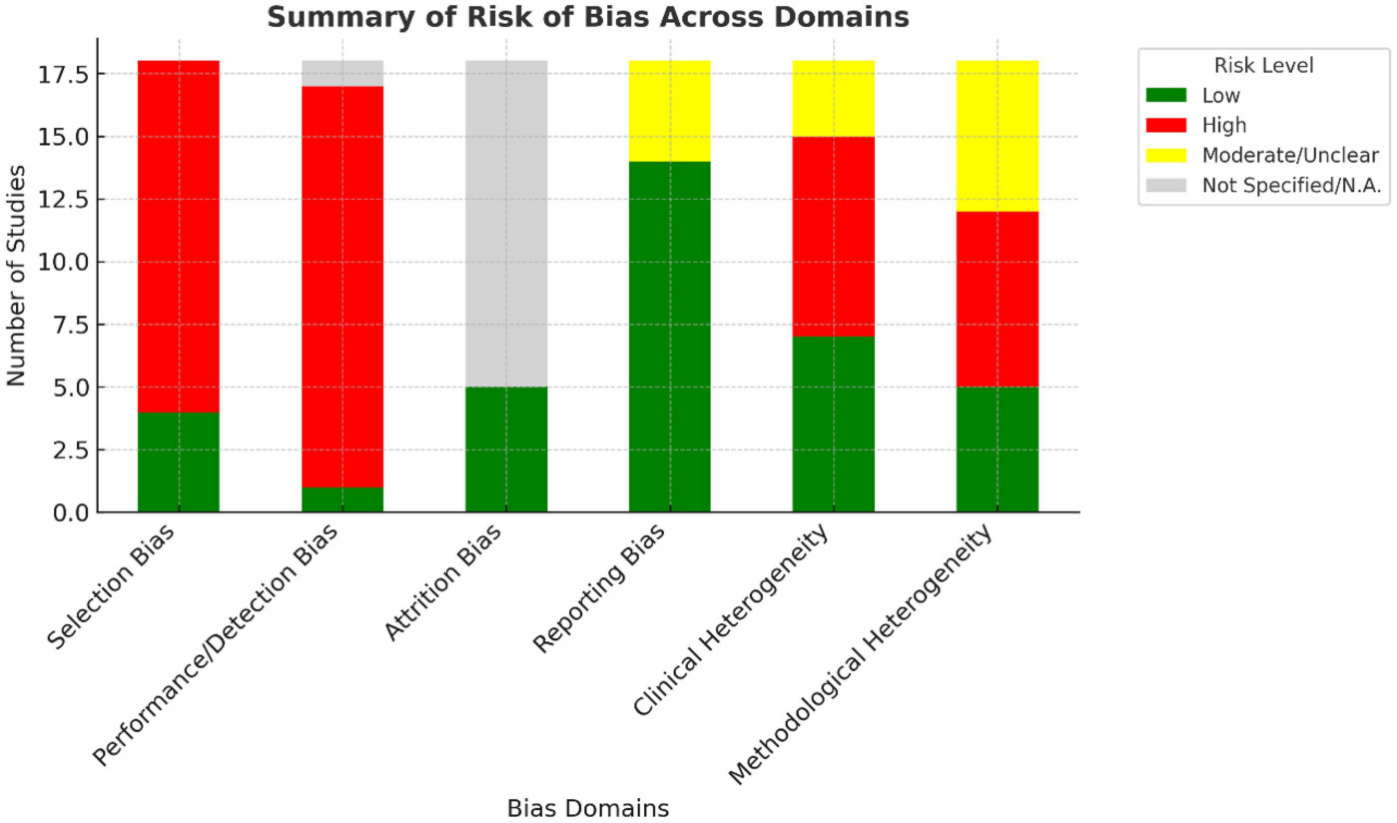
Summary of risk of bias across included studies.

The funnel plot in Figure 5 helps assess potential publication bias in the meta-analysis. The funnel plot shows the relationship between standard error and Cohen’s *d*. Ideally, a funnel-shaped plot would indicate that small and large studies are represented symmetrically.

**Figure 6:**
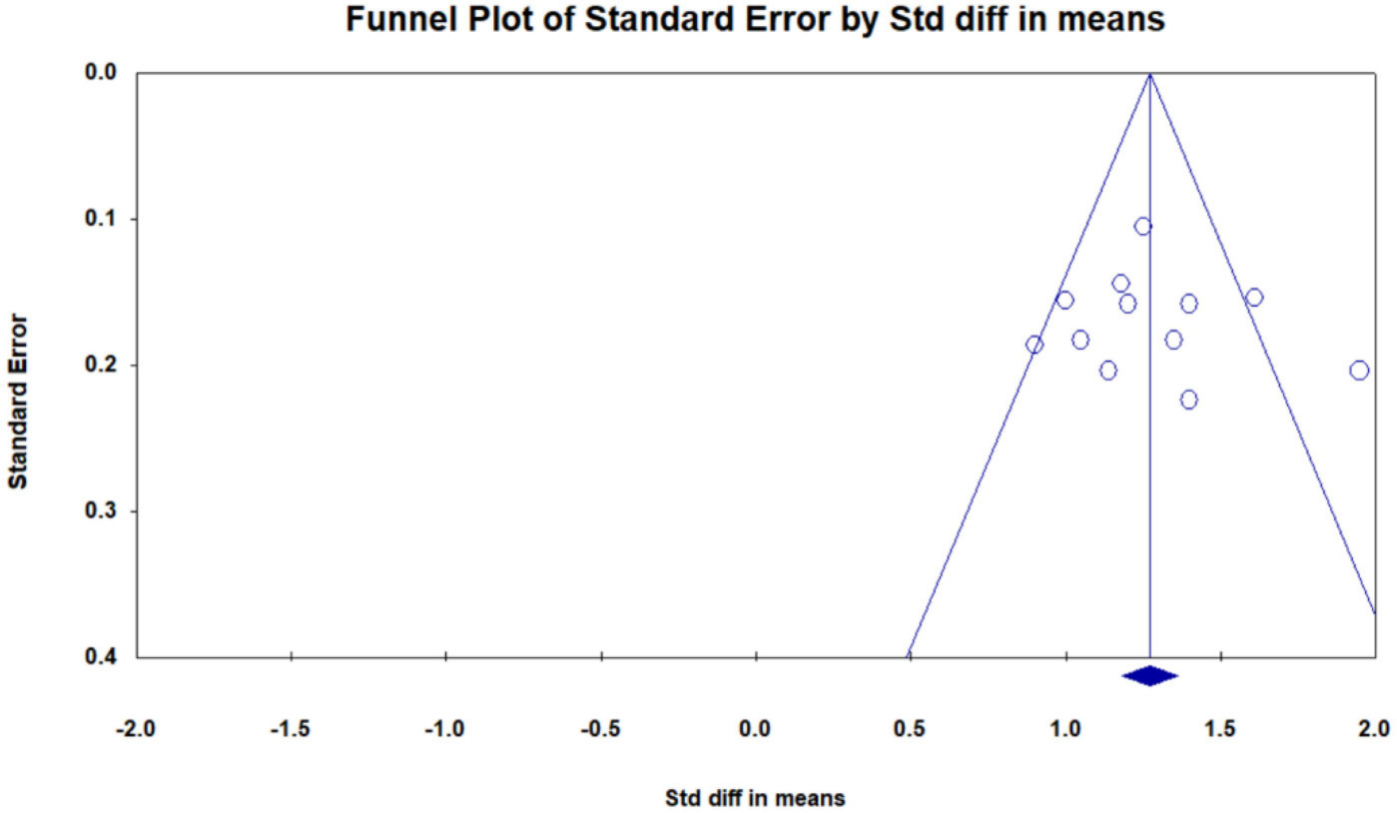
Funnel plot of standard error vs. standardised mean difference (Cohen’s *d*) for studies included in the meta-analysis.

Figure 6 shows the distribution of the studies, with most studies clustered around a Cohen’s *d* of 1.0. The asymmetry in the plot suggests a slight publication bias, with more studies reporting positive or significant effects than negative or non-significant effects. However, the plot appears reasonably symmetrical overall, suggesting publication bias may not be a major concern for this analysis.

## Discussion

The present systematic review and meta-analysis synthesised evidence from 12 studies examining the effectiveness of physical activity (PA) interventions for children and adolescents with autism spectrum disorder (ASD). The findings demonstrate that PA interventions produce multidomain improvements, with the strongest effects observed in motor and physical fitness outcomes, followed by social and communication domains, while behavioural outcomes showed comparatively smaller effects. Evidence from structured motor interventions such as school-based games and ball training consistently demonstrated moderate to large improvements (37, 49), whereas behavioural outcomes, including reductions in irritability and stereotypy, showed smaller but meaningful effects (55). The pooled effect size (*d* = 1.278) indicates a large overall intervention effect, although moderate heterogeneity (*I*^2^ = 58.67%) suggests variability in intervention impact across studies. While these findings were statistically significant, statistical significance indicates that the observed effects were unlikely to have occurred by chance and do not automatically confirm meaningful real-world or clinical impact. Clinical significance depends on whether the improvements are substantial enough to enhance everyday functioning, participation, adaptive behaviour, or quality of life for children with ASD. Therefore, effect sizes should be interpreted alongside the practical relevance of the outcomes achieved. Importantly, all reported effects reflect adjusted outcomes controlling for baseline differences where applicable, thereby strengthening the internal validity of the findings.

### Characteristics of physical activity interventions

The analysis revealed substantial diversity in the characteristics of PA interventions, reflecting both innovation (37, 53) and inconsistency (60). Interventions ranged from structured sport-based programmes such as mini-basketball (46, 51) and adapted judo (47) to multimodal approaches integrating sensory, cognitive, and social components (37, 54, 58).

Duration and implementation varied considerably. Some interventions were delivered over short-term periods, particularly feasibility and pre–post designs (43, 57), while others were embedded within longer, structured programmes such as institutional or school-based interventions (46, 52, 53). This variation reflects both adaptation to contextual constraints and a lack of standardised intervention frameworks. This variation not only reflects contextual adaptation but also introduces inconsistencies in dosage, intensity, and exposure, which may partially explain variability in effect sizes observed across studies.

Furthermore, inconsistent reporting of fidelity, as observed in Milajerdi et al. (45), Chen et al. (50), Qi et al. (51), Mao et al. (52), Wu and Cai (53), Wen and Wu (37), Coffey et al. (55), and Zhang et al. (56), limits confidence in whether interventions were delivered as intended, thereby affecting internal validity. This limitation is particularly critical, as fidelity directly influences reproducibility, scalability, and the translation of interventions into real-world educational and clinical settings.

### Effects across multidomain outcomes

The findings indicate that PA interventions are effective across multiple developmental domains, although the magnitude of effects varies systematically. Motor and physical fitness outcomes demonstrated the greatest and most consistent improvements, particularly in structured sport interventions such as ball-skill training and coordinated exercise programmes (37, 49, 52). These effects are likely driven by the direct targeting of neuromuscular coordination, balance, and strength, which respond rapidly to repetitive and structured movement-based training (14, 62). This aligns with theoretical expectations that motor systems respond more directly and rapidly to structured, repetitive physical training compared to higher-order cognitive or behavioural domains.

Social and communication outcomes also improved substantially, especially in group-based interventions. Programmes such as mini-basketball and combined ball training (46, 51) and integrated social sport models (54) demonstrated significant gains in social cognition, communication, and engagement. These improvements can be attributed to the embedded social demands of such interventions, including turn-taking, shared attention, and cooperative interaction. These findings support the premise that social interaction embedded within physical activity contexts serves as a naturalistic mechanism for enhancing communication and peer-related skills.

Executive functioning outcomes showed emerging improvements, though less consistent. Structured interventions that incorporate cognitive demands, such as rule-based games and coordinated sports activities, demonstrated gains in inhibitory control and cognitive flexibility (50), whereas other studies reported limited or domain-specific effects (56). This suggests that executive outcomes are sensitive to intervention design, particularly the extent to which cognitive engagement is explicitly integrated.

Behavioural outcomes showed smaller effect sizes, with improvements primarily observed in irritability, social withdrawal, and stereotypy (55). The relatively smaller magnitude of behavioural change may reflect the multifactorial nature of behavioural regulation in ASD, which often requires combined behavioural, environmental, and therapeutic approaches beyond physical activity interventions alone.

### Magnitude of intervention effects (meta-analytic findings)

The pooled effect size (*d* = 1.278) indicates a large overall effect of PA interventions, reinforcing their potential as effective intervention strategies. However, statistical significance should not be equated with clinical significance, which requires consideration of the magnitude and practical importance of changes in real-life functioning. Several studies contributed to large individual effect sizes, particularly those employing structured and multimodal approaches, such as Wu and Cai (53), which reported substantial improvements in joint attention (d up to 2.7), and Xing et al. (54), which demonstrated large gains in both physical activity and social engagement.

However, the magnitude of these effects exceeds those reported in earlier meta-analyses (11, 12), suggesting the need for cautious interpretation. This discrepancy may be partially attributed to the inclusion of non-randomised and pre–post designs, which are more susceptible to bias and may inflate effect estimates, as well as the increasing use of multimodal interventions that target multiple domains simultaneously. Furthermore, the large, pooled effect size should be interpreted in light of the observed heterogeneity and potential small-study effects, which may contribute to overestimation of intervention impact.

### Methodological quality and risk of bias

The methodological quality of included studies presents several limitations. Many studies relied on small samples, such as those by Ferreira et al. (57) and Dehghani et al. (48), thereby reducing statistical power and increasing susceptibility to random error. The frequent use of convenience sampling further limits generalisability. Blinding procedures were rarely implemented, increasing the risk of performance and detection bias, particularly for subjective outcomes such as behavioural and social measures (63).

In addition, outcome measures varied widely across studies, with some relying on standardised tools (e.g., BOTMP, SRS-2) and others using researcher-developed instruments (43, 56), reducing comparability. The inconsistent use of validated measures introduces measurement bias and limits confidence in cross-study synthesis. Moreover, the lack of consistent reporting of intervention fidelity further weakens internal validity, as it remains unclear whether observed effects are attributable to intervention content or variations in implementation. These methodological limitations highlight the need for more rigorous and standardised research designs.

### Sources of heterogeneity across studies

The moderate heterogeneity observed (*I*^2^ = 58.67%) reflects variability across intervention, participant, and methodological factors. Intervention diversity, including differences in type, duration, and intensity, contributed significantly to variability (6, 37). Participant characteristics also played a role. Studies included a wide age range, from preschool children (51) to adolescents (57), and varied in ASD severity and comorbidity profiles (58). Such differences influence baseline functioning and responsiveness to intervention. Methodological diversity further contributed to heterogeneity.

The inclusion of RCTs, quasi-experimental, and pre–post designs introduces variability in effect estimation, as non-randomised designs are more prone to bias (60). Differences in outcome measures also limit comparability across studies. Subgroup analyses by study design, intervention type, and age group further confirm that heterogeneity is not random but systematically linked to these factors, reinforcing the importance of stratified interpretation of results.

### Publication bias and effect size considerations

The funnel plot analysis suggests slight asymmetry, indicating the potential presence of publication bias or small-study effects. Smaller studies, such as those with limited sample sizes [e.g. (48)], tended to report larger effects, potentially inflating pooled estimates. Additionally, several effect sizes were derived from secondary statistics, such as eta-squared, rather than directly reported values (49, 50), introducing potential estimation errors. This reliance on derived effect sizes may reduce precision and contribute to variability in pooled estimates. As noted by Reeves et al. (64), combining heterogeneous designs and derived effect sizes may lead to overestimation. The magnitude of the pooled estimate should therefore not be interpreted as definitive evidence of uniformly large intervention effects, but rather as a summary estimate influenced by study quality, sample size, and reporting characteristics.

### Implications for practice and future research

The findings support integrating PA interventions across educational, clinical, and community settings. Structured programmes such as mini-basketball (46) and multimodal sport interventions (54) demonstrate clear benefits for motor, social, and cognitive outcomes. These findings translate directly into classroom practice by providing teachers with a structured, low-cost framework for improving attention, engagement, and participation among learners with ASD through movement-based activities embedded in daily routines. Importantly, interventions that combine physical, cognitive, and social elements appear to yield the most comprehensive benefits. In resource-limited contexts, such programmes offer cost-effective and scalable solutions for supporting children with ASD.

Future research should prioritise large-scale, well-designed randomised controlled trials to strengthen the evidence base and reduce bias. Standardised outcome measures are needed to improve comparability across studies and enhance the precision of meta-analytic findings. Longitudinal studies are essential to assess the durability of intervention effects, as current evidence is largely limited to short-term outcomes. Greater attention should also be given to intervention fidelity and implementation processes to enhance reproducibility.

Greater emphasis should also be placed on fidelity monitoring, implementation science frameworks, and real-world applicability to ensure that interventions can be effectively scaled and sustained across diverse contexts. Additionally, future studies should include more diverse populations, particularly females and underrepresented groups, and explore subgroup differences based on age, ASD severity, and intervention characteristics. Such analyses would provide more nuanced insights into the conditions under which PA interventions are most effective.

## Conclusion

This systematic review and meta-analysis provide comprehensive evidence that physical activity (PA) interventions are associated with meaningful improvements across multiple developmental domains in children and adolescents with autism spectrum disorder (ASD). The strongest and most consistent effects were observed in motor skills and physical fitness, reflecting the alignment between intervention design and physiological outcomes. Substantial gains were also identified in social communication, particularly in group-based and structured programmes that facilitate interaction and engagement. Improvements in executive functioning were observed in cognitively enriched interventions, while behavioural outcomes showed more modest but still relevant changes.

The pooled effect size indicates a large association between PA interventions and developmental outcomes; however, this estimate should be interpreted with caution. Variability in study design, intervention characteristics, and outcome measurement, alongside methodological limitations such as small sample sizes, lack of blinding, and inconsistent fidelity reporting, may influence the strength and stability of these associations. These findings highlight both the promise and the current limitations of the evidence base.

Multimodal PA interventions that integrate physical, cognitive, and social components appear to be linked with broader developmental benefits in children with ASD. Future research should prioritise rigorous experimental designs, standardised outcome measures, and longitudinal evaluation to strengthen the evidence base and enhance reproducibility. Advancing this field requires not only methodological refinement but also a sustained commitment to developing scalable, inclusive, and contextually relevant interventions that can support improved outcomes for individuals with ASD.

## Data Availability

The original contributions presented in the study are included in the article/Supplementary Material, further inquiries can be directed to the corresponding author.
