# Supplementary material for: Sports and physical activity interventions in autism spectrum disorder: a systematic literature review and meta-analysis
Source: Front Sports Act Living. 2026 Jun 26;8:1793290. doi: 10.3389/fspor.2026.1793290 (PMC13350350; doi:10.3389/fspor.2026.1793290)
Supplement: Supplementary file 1 [file Datasheet1.pdf]

Appendix A

Supplementary Material S1: Detailed Search Strategy

A1. Database and Search Parameters

The electronic search was conducted in **PubMed** to identify studies examining physical activity interventions in children and adolescents with autism spectrum disorder (ASD). The search was conducted in March 2025 and included all records available as of the search date. Searches were conducted using a combination of Medical Subject Headings (MeSH) and free-text terms to ensure comprehensive coverage.

The search was limited to:

- Human participants
- English-language publications

No date restrictions were applied during the initial search phase.

A2. Search Strategy Structure

The search strategy was developed around three core conceptual domains:

1. **Population:** Autism spectrum disorder
2. **Intervention:** Physical activity, exercise, and sport
3. **Outcomes:** Motor, social, executive, behavioural, and psychosocial domains

These domains were combined using Boolean operators (AND, OR) to maximise sensitivity and specificity.

A3. Full PubMed Search String

The final PubMed search string was as follows:

(("Autism Spectrum Disorder"[Mesh] OR "autism spectrum disorder"[tiab] OR ASD[tiab] OR autism[tiab])

AND

("Motor Activity"[Mesh] OR "Exercise"[Mesh] OR "Sports"[Mesh]

OR "physical activity"[tiab] OR exercise[tiab] OR sport*[tiab]

OR movement[tiab] OR training[tiab] OR aerobic[tiab]

OR "physical fitness"[tiab] OR "motor training"[tiab])

AND

("Motor Skills"[Mesh] OR "Social Skills"[Mesh] OR "Executive Function"[Mesh]

OR "motor skill*"[tiab] OR coordination[tiab] OR balance[tiab]

OR "social skill*"[tiab] OR communication[tiab]

OR "executive function*"[tiab] OR cognition[tiab]

OR behavior[tiab] OR behavioural[tiab] OR psychosocial[tiab]))

A4. Adaptation Across Databases

The PubMed search strategy served as the reference template and was adapted for use in **Scopus, PsycINFO, SPORTDiscus, and ERIC**. Adjustments were made to account for differences in indexing systems, subject headings, and search syntax across databases. Equivalent keywords and controlled vocabulary terms were used where applicable.

A5. Additional Search Procedures

To enhance coverage and minimise publication bias, the following supplementary search procedures were undertaken:

- Manual screening of reference lists of included studies
- Screening of relevant systematic reviews
- Searches of trial registries and journal sources

No additional eligible studies were identified through these procedures.

A6. Reproducibility Statement

The search strategy was designed to ensure transparency, reproducibility, and methodological rigour. The inclusion of both controlled vocabulary (MeSH terms) and free-text keywords aimed

to maximise the retrieval of relevant studies while maintaining specificity. This detailed reporting aligns with **PRISMA 2020 recommendations for systematic reviews**.
